# Reply to Dunican, I.C.; Walsh, J.H. Comment on “Gratwicke et al. Nutritional Interventions to Improve Sleep in Team-Sport Athletes: A Narrative Review. *Nutrients* 2021, *13*, 1586”

**DOI:** 10.3390/nu13093104

**Published:** 2021-09-03

**Authors:** Madeleine Gratwicke, Kathleen H. Miles, David B. Pyne, Kate L. Pumpa, Brad Clark

**Affiliations:** 1 Research Institute for Sport and Exercise, University of Canberra, Canberra 2617, Australia; maddy.gratwicke@canberra.edu.au (M.G.); kathleen.miles@canberra.edu.au (K.H.M.); David.Pyne@canberra.edu.au (D.B.P.); Kate.Pumpa@canberra.edu.au (K.L.P.); 2 Discipline of Sport and Exercise Science, University of Canberra, Canberra 2617, Australia

We thank Dunican and Walsh [1] for taking the time to read and comment on the sleep-nutrition review. First, they have rightly identified an editing error in our article where we have erroneously indicated that tart cherry juice had been shown to enhance sleep indices as assessed by polysomnography (PSG). The in-text citations were in the incorrect order. We should have stated that tart cherry juice enhanced sleep indices as assessed by sleep questionnaires, and wrist actigraphy monitoring, in healthy individuals without sleep problems [2], and in individuals with sleep problems such as insomnia [3]. We apologise for this error and take this opportunity to correct the record.

While PSG was not used in either study [2,3], both studies report consistent improvement in sleep indices in their sample. The changes in total sleep time (TST) described by Howatson and colleagues [2] are particularly compelling given they are based on actigraphy which has been shown to accurately measure TST against PSG [4,5]. We acknowledge that PSG is the gold standard measure of sleep quality and quantity, and reiterate the call made in our review for further research on the effects of nutrients on PSG-measured sleep. However, actigraphy represents a more feasible sleep monitoring tool for use in sport and applied research settings where access to the requisite equipment, expertise and time often excludes the use of PSG. Where studies consistently report that actigraphy-monitored sleep is stable or improves following nutrient ingestion, scientists, coaches and athletes can reasonably consider including that nutrient or nutrients in the appropriate context to support sleep and recovery.

Dunican and Walsh [1] query the link between folate deficiency and restless leg syndrome. The review paper by Kelly [6] we cited in our review describes early research that demonstrates a link between folate deficiency and restless leg syndrome, as well as resolution of restless leg syndrome following folate supplementation in neurological conditions [7,8,9]. This link is made in other reviews on restless leg syndrome [10], and of the effect of diet on sleep [11]. However, we agree that further research is required to determine if there is any clinically relevant link between folate deficiency and restless leg syndrome in athlete populations.

Dunican and Walsh [1] also suggest the strength of evidence provided by studies included in our review excludes their use for practical recommendations. As the review was a narrative synthesis, we did not complete a formal quantitative analysis of the quality of the included studies. However, we respectfully disagree with the sentiments expressed by the correspondents regarding the quality of evidence provided by the studies. Of the 19 studies described by our review, only three [12,13,14] did not compare the relevant nutrient with a placebo or control condition, only two studies [12,14] used a non-randomised study design, only four did not clearly indicate use of single or double blinding [12,13,14,15], and only five did not use an objective measure of sleep [2,15,16,17]. Furthermore, for most specific nutrients or foods, there was at least one study that used a randomized design, included a control or placebo condition, and employed an objective measure of sleep. The only exception to these conditions was beetroot for which there was one study [15] that used a randomized controlled and placebo-controlled trial, but did not blind participants or researchers, and only included subjective measures of sleep. Accordingly, the strength of evidence for most nutrients is almost certainly not “weak”, and taken together, lends support to use of certain nutrients to promote sleep in sport settings. However, further well-designed and appropriately powered studies are required to confirm the efficacy for many nutrients. Hence, we contextualized the synthesis of studies in this review with extensive calls for further research in both the Discussion and Conclusion.

Finally, we sought to review papers describing nutrients linked to enhanced sleep indices. Studies describing the effects of substances linked to impaired sleep such as caffeine and alcohol were beyond the scope of the review. However, we recognize the strength of evidence for avoiding these substances, and for completeness highlighted selected nutrients, recommending athletes avoid them prior to sleep. 

## Data Availability

Not applicable.
